# Supplementary material for: A mapping review of worldwide current and previous cohort research programmes in cats and dogs
Source: PLoS One. 2025 Jun 2;20(6):e0321007. doi: 10.1371/journal.pone.0321007 (PMC12129338; doi:10.1371/journal.pone.0321007)
Supplement: S1 File — (DOCX) [file pone.0321007.s001.docx]

**S1 File. Search string and keywords used in Rayya in the mapping review of cats and dogs research cohort programmes.**

**Embase.com**

| No.,Query,Results,Date |
| --- |
| #8,"('data collection method'/exp AND ('pet animal'/exp OR 'dog'/exp OR 'cat'/exp) AND ('cohort analysis'/exp OR 'longitudinal study'/exp OR 'prospective study'/exp) OR (((dogs OR dog OR 'canis familiaris' OR 'canis lupus familiaris' OR 'c familiaris' OR 'c lupus familiaris' OR puppy OR puppies OR canine OR cat OR cats OR 'felis catus' OR 'f catus' OR 'felis domestica' OR 'felis domesticus' OR 'felis sylvestris catus' OR felix OR feline OR felines OR kitten OR kittens) NEAR/5 (cohort OR longitudinal OR prospective)):ti,ab) OR ((dog:kw OR dogs:kw OR canine:kw OR cat:kw OR cats:kw OR feline:kw) AND (cohort:kw OR longitudinal:kw OR prospective:kw))) NOT ('conference paper':it OR 'conference review':it)",4704,19 Dec 2023 |

**Ovid MEDLINE(R)** and Epub Ahead of Print, In-Process, In-Data-Review & Other Non-Indexed Citations and Daily <1946 to December 18, 2023>

1 ((exp "Surveys and Questionnaires"/ or clinical protocols/) and (cohort studies/ or longitudinal studies/ or prospective studies/) and (dogs/ or cats/ or pets/)) or ((dogs or dog or "Canis familiaris" or "canis lupus familiaris" or "c familiaris" or "c lupus familiaris" or puppy or puppies or canine or Cat or Cats or "Felis catus" or "F catus" or "Felis domestica" or "Felis domesticus" or "Felis sylvestris catus" or Felix or Feline or felines or Kitten or kittens) adj5 (cohort or longitudinal or prospective)).ti,ab. or ((dog or dogs or canine or cat or cats or feline) and (cohort or longitudinal or prospective)).kf. 2668

# **Web of Science Search Strategy** (v0.1)

# Database: Web of Science Core Collection

# Entitlements:

- WOS.IC: 1993 to 2023

- WOS.CCR: 1985 to 2023

- WOS.SCI: 1900 to 2023

- WOS.AHCI: 1975 to 2023

- WOS.BHCI: 2005 to 2023

- WOS.BSCI: 2005 to 2023

- WOS.ESCI: 2018 to 2023

- WOS.ISTP: 1990 to 2023

- WOS.SSCI: 1900 to 2023

- WOS.ISSHP: 1990 to 2023

# Searches:

6: (TI=((dogs OR dog OR "Canis familiaris" OR "canis lupus familiaris" OR "c familiaris" OR "c lupus familiaris" OR puppy OR puppies OR canine OR Cat OR Cats OR "Felis catus" OR "F catus" OR "Felis domestica" OR "Felis domesticus" OR "Felis sylvestris catus" OR Felix OR Feline OR felines OR Kitten OR kittens) NEAR/6 (cohort OR longitudinal OR prospective)) OR AB=((dogs OR dog OR "Canis familiaris" OR "canis lupus familiaris" OR "c familiaris" OR "c lupus familiaris" OR puppy OR puppies OR canine OR Cat OR Cats OR "Felis catus" OR "F catus" OR "Felis domestica" OR "Felis domesticus" OR "Felis sylvestris catus" OR Felix OR Feline OR felines OR Kitten OR kittens) NEAR/6 (cohort OR longitudinal OR prospective)) OR AK=((dog OR dogs OR canine OR cat OR cats OR feline) AND (cohort OR longitudinal OR prospective))) NOT CF=(dog OR dogs OR cat OR cats OR cohort OR longitudinal OR prospective) Date Run: Tue Dec 19 2023 14:40:00 GMT+0100 (Mitteleuropäische Normalzeit) Results: 3611

**Scopus**

19.12.23, 5341

(TITLE-ABS((dogs OR dog OR "Canis familiaris" OR "canis lupus familiaris" OR "c familiaris" OR "c lupus familiaris" OR puppy OR puppies OR canine OR Cat OR Cats OR "Felis catus" OR "F catus" OR "Felis domestica" OR "Felis domesticus" OR "Felis sylvestris catus" OR Felix OR Feline OR felines OR Kitten OR kittens) W/6 (cohort OR longitudinal OR prospective)) OR AUTHKEY((dog OR dogs OR canine OR cat OR cats OR feline) AND (cohort OR longitudinal OR prospective))) AND NOT (DOCTYPE ( cp OR cr ))

**Keywords used in Rayyan**

Keywords used in Rayyan for *inclusion were*: animal, animals, cohort, prevalence, prospective, longitudinal, observational, companion, dogs, cats, canine;

*and for exclusion*: randomized, cells, trial, randomly, controlled, placebo, review, vitro, human, retrospective, retrospectively, cross-sectional, case-control.
