# Supplementary material for: A mapping review of worldwide current and previous cohort research programmes in cats and dogs
Source: PLoS One. 2025 Jun 2;20(6):e0321007. doi: 10.1371/journal.pone.0321007 (PMC12129338; doi:10.1371/journal.pone.0321007)
Supplement: S3 File — Ettinger and Edward C. Feldman. (DOCX) [file pone.0321007.s003.docx]

### **S3 File. List of the veterinary research specialty(s) according to the Textbook of Small Animal Internal Medicine - 8th Edition by Stephen J. Ettinger and Edward C. Feldman**

**Volume 1:**

1. **Introduction to Internal Medicine**
2. **Clinical Approach to the Patient**
3. **Laboratory Diagnostics**
4. **Behavioral Disorders**
5. **Cardiovascular System**
6. **Respiratory System**
7. **Gastrointestinal System**
8. **Hepatobiliary System**
9. **Renal System**
10. **Endocrine System**
11. **Hematology**
12. **Immune System**

**Volume 2:**

1. **Neurology**
2. **Dermatology**
3. **Oncology**
4. **Infectious Diseases**
5. **Reproductive System**
6. **Emergency and Critical Care**
7. **Geriatrics**
8. **Preventive Medicine and Public Health**
9. **Clinical Pharmacology**
