# Supplementary figures and images for: A mapping review of worldwide current and previous cohort research programmes in cats and dogs
Source: PLoS One. 2025 Jun 2;20(6):e0321007. doi: 10.1371/journal.pone.0321007 (PMC12129338; doi:10.1371/journal.pone.0321007)

# Start of enrollmnet

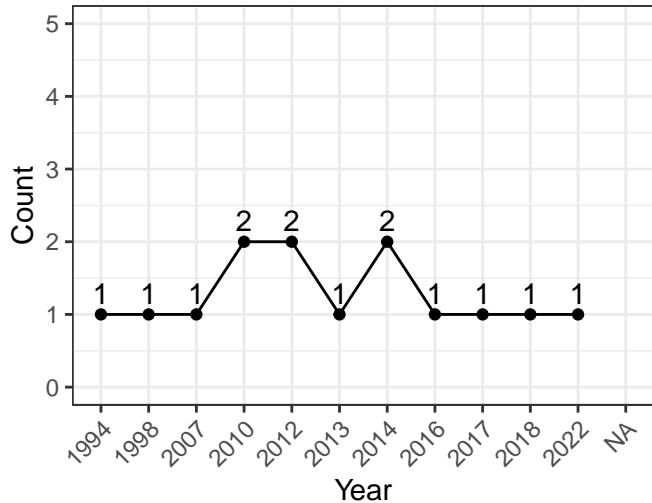

# First publication

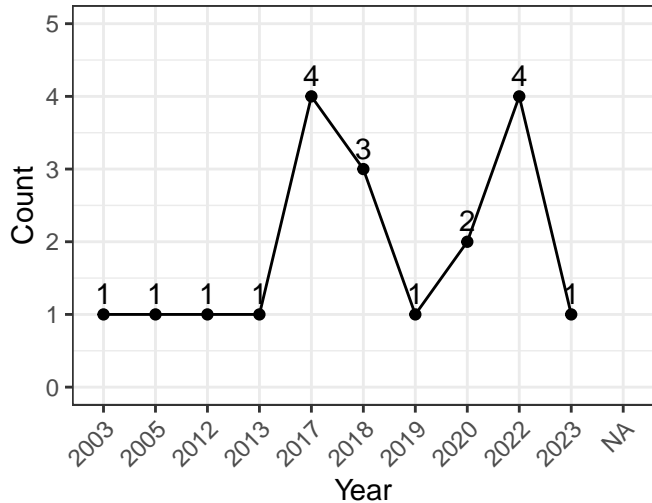

Supplement: S1 Fig — (PDF) [file pone.0321007.s004.pdf]

Figure S2. Data sources and promotion methods

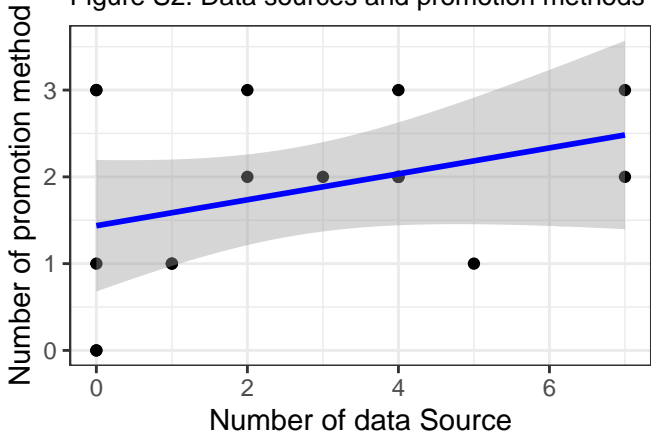

Supplement: S2 Fig — (PDF) [file pone.0321007.s005.pdf]
