# Supplementary material for: A mapping review of worldwide current and previous cohort research programmes in cats and dogs
Source: PLoS One. 2025 Jun 2;20(6):e0321007. doi: 10.1371/journal.pone.0321007 (PMC12129338; doi:10.1371/journal.pone.0321007)
Supplement: S1 Table — This table shows the age limit used for inclusion or exclusion in the programs. For 9 it was a criterion and for another 5 it was not a criterion whether the animal could participate in the program or not. However, for 3 programs, there was no information available regarding whether age was a criterion. (DOCX) [file pone.0321007.s006.docx]

**S1 Table.**  **Detailed age inclusion/exclusion criteria across 19 programmes in the mapping review of cats and dogs cohort research programmes.**

| **Species** | **Age Distribution** | **Number of Programmes** |
| --- | --- | --- |
| **Cats** |  |  |
|  | 2-4 months | 1 |
|  | 7-9 years | 1 |
|  | 8-24 weeks | 1 |
| **Dogs** |  |  |
|  | <16 weeks | 1 |
|  | <16 weeks / <21 weeks | 1 |
|  | >6 months, <2 years | 1 |
|  | ≥18 months | 1 |
|  | 6-8 weeks | 1 |
|  | Inclusion when bitch was mated | 1 |
|  | Not applicable (NA) | 3 |
| **Cat and Dogs** | ≥ 6 months, <10 years (dogs),  <12 years (cat)s | 1 |
|  | Not applicable | 2 |

*Legend:* This table shows the age limit used for inclusion or exclusion in the programs. For 9 it was a criterion and for another 5 it was not a criterion whether the animal could participate in the program or not. However, for 3 programs, there was no information available regarding whether age was a criterion.
