# Supplementary material for: A mapping review of worldwide current and previous cohort research programmes in cats and dogs
Source: PLoS One. 2025 Jun 2;20(6):e0321007. doi: 10.1371/journal.pone.0321007 (PMC12129338; doi:10.1371/journal.pone.0321007)
Supplement: S2 Table — (DOCX) [file pone.0321007.s007.docx]

**S2 Table.** **Considered outcomes in cat and dog cohort research programmes.**

| **ID** | **Programme name** | **Paper title** | **year** | **DOI** | **Outcomes**  **total** | **Outcome**  **1** | **Outcome2** | **Outcome**  **3** | **Outcome**  **4** |
| --- | --- | --- | --- | --- | --- | --- | --- | --- | --- |
| 1 | Generation Pup | Like Living with a Sassy Teenager!â€: A Mixed-Methods Analysis of Ownersâ€™ Comments about Dogs between the Ages of 12 Weeks and 2 Years | 2023 | 10.3390/ani13111863 | 4 | Owners’ Attributions of Dog Behaviour | Change in Dog Behaviour | Positive Experiences Related to Dog Ownership | Negative Experiences Related to Dog Ownership |
| 1 | Generation Pup | Dog walk frequency and duration: Analysis of a cohort of dogs up to 15 months of age | 2022 | 10.1016/j.applanim.2022.105609 | 4 | walk frequency | walk duration | Proportion of walk spent on lead |  |
| 1 | Generation Pup | Generation Pup' â€“ protocol for a longitudinal study of dog behaviour and health | 2021 | 10.1186/s12917-020-02730-8 | 0 | methodological paper |  |  |  |
| 1 | Generation Pup | Factors influencing owner-reported approaches to training dogs enrolled in the Generation Pup longitudinal study | 2021 | 10.1016/j.applanim.2021.105404 | 1 | Reported training approaches |  |  |  |
| 1 | Generation Pup | Owner perception of problem behaviours in dogs aged 6 and 9-months | 2020 | 10.1016/j.applanim.2020.105147 | 1 | Owners reporting behavioural problem |  |  |  |
| 1 | Generation Pup | Birth cohort study of pet dogs underway and recruiting | 2017 | 10.1136/vr.j3495 | 0 | methodological paper |  |  |  |
| 2 | Dog Aging Project | An open science study of ageing in companion dogs | 2022 | 10.1038/s41586-021-04282-9 | 8 | cause of death | age of death |  |  |
| 2 | Dog Aging Project | Big data from small animals: integrating multi-level environmental data into the Dog Aging Project | 2023 | 10.20506/rst.42.3349 | 0 | methodological paper |  |  |  |
| 2 | Dog Aging Project | Development and validation of a novel instrument to capture companion dog mortality data: the Dog Aging Project End of Life Survey | 2023 | 10.2460/javma.23.02.0078 | 0 | methodological paper |  |  |  |
| 2 | Dog Aging Project | Social determinants of health and disease in companion dogs: A cohort study from the Dog Aging Project | 2023 | 10.1093/emph/eoad011 | 1 | Health outcomes |  |  |  |
| 2 | Dog Aging Project | Lifetime prevalence of owner-reported medical conditions in the 25 most common dog breeds in the Dog Aging Project pack | 2023 | <https://dx.doi.org/10.3389/fvets.2023.1140417> | 3 | Seasonal allergies | Ear infection |  |  |
| 2 | Dog Aging Project | Evaluation of Cognitive Function in the Dog Aging Project: Associations with Baseline Canine Characteristics | 2022 | 10.1101/2022.05.04.490636 | 1 | canine cognitive dysfunction |  |  |  |
| 2 | Dog Aging Project | LIFETIME PREVALENCE OF TUMORS IN COMPANION DOGS INCLUDED IN THE DOG AGING PROJECT BASELINE DATA | 2022 | 10.1111/jvim.16538 | 2 | Malignant tumour diagnosis | Benign tumour diagnosis |  |  |
| 2 | Dog Aging Project | Purpose, Partnership, and Possibilities: The Implementation of the Dog Aging Project Biobank | 2022 | 10.1177/11772719221137217 | 4 | defining an aged dog phenotype | identify genetic variation | aging | Effect of Rapamycin in aging dogs |
| 2 | Dog Aging Project | Demographic factors associated with joint supplement use in dogs from the Dog Aging Project | 2022 | 10.3389/fvets.2022.906521 | 1 | administration of joint supplements |  |  |  |
| 2 | Dog Aging Project | Associations between physical activity and cognitive dysfunction in older companion dogs: Results from the Dog Aging Project | 2022 | 10.1101/2022.04.20.488879 | 1 | canine cognitive dysfunction |  |  |  |
| 2 | Dog Aging Project | THE LINK BETWEEN ENVIRONMENT, AGE, AND HEALTH IN A LARGE COHORT OF COMPANION DOGS FROM THE DOG AGING PROJECT | 2021 |  | 3 | health | disease | mobility |  |
| 3 | Golden Retriver Lifetime Study | Cohort profile: The Golden Retriever Lifetime Study (GRLS) | 2022 | 10.1371/journal.pone.0269425 | 0 | methodological paper |  |  |  |
| 3 | Golden Retriver Lifetime Study | HEMANGIOSARCOMA IN THE GOLDEN RETRIEVER LIFETIME STUDY COHORT: MORRIS ANIMAL FOUNDATION'S GRAND CHALLENGE | 2023 | 10.1111/vco.12867 | 1 | Hemangiosarcoma |  |  |  |
| 3 | Golden Retriver Lifetime Study | Environmental exposures and lymphoma risk: a nested case-control study using the Golden Retriever Lifetime Study cohort | 2022 | <https://dx.doi.org/10.1186/s40575-022-00122-9> | 1 | Lymphma |  |  |  |
| 3 | Golden Retriver Lifetime Study | The Golden Retriever Lifetime Study: Assessing factors associated with owner compliance after the first year of enrollment | 2021 | 10.1111/jvim.15921 | 1 | owner compliance after first year of enrollement |  |  |  |
| 4 | VetCompass Australia | VetCompass Australia: A National Big Data Collection System for Veterinary Science | 2017 |  | 0 | methodological paper |  |  |  |
| 4 | VetCompass Australia | Prednisolone prescribing practices for dogs in Australia | 2023 | 10.1371/journal.pone.0282440 | 1 | Prednisolone prescribing practices for dogs in Australia |  |  |  |
| 5 | VetCompass | Leptospirosis vaccination in dogs attending UK primary care practices: vaccine uptake and factors associated with administration | 2022 | 10.1186/s12917-022-03382-6 | 1 | Leptospirosis vaccination annual prevalence |  |  |  |
| 5 | VetCompass | Breed and conformational predispositions for prolapsed nictitating membrane gland (PNMG) in dogs in the UK: A VetCompass study | 2022 | 10.1371/journal.pone.0260538 | 1 | Prolapsed nictitating membrane gland (PNMG) annual prevalence |  |  |  |
| 5 | VetCompass | Pandemic Puppies: Demographic Characteristics, Health and Early Life Experiences of Puppies Acquired during the 2020 Phase of the COVID-19 Pandemic in the UK | 2022 | 10.3390/ani12050629 | 1 | Effects of COVID-19 Pandemic on Puppies |  |  |  |
| 5 | VetCompass | Occurrence and clinical management of urethral obstruction in male cats under primary veterinary care in the United Kingdom in 2016 | 2022 | 10.1111/jvim.16389 | 1 | urethral obstruction male cats |  |  |  |
| 5 | VetCompass | Frequency and predisposing factors for canine otitis externa in the UK - a primary veterinary care epidemiological view | 2021 | <https://dx.doi.org/10.1186/s40575-021-00106-1> | 1 | canine otitis externa |  |  |  |
| 5 | VetCompass | Epidemiology of periodontal disease in dogs in the UK primary-care veterinary setting | 2021 | 10.1111/jsap.13405 | 1 | periodontal disease |  |  |  |
| 5 | VetCompass | Reporting the epidemiology of aural haematoma in dogs and proposing a novel aetiopathogenetic pathway | 2021 | 10.1038/s41598-021-00352-0 | 1 | aural haematoma |  |  |  |
| 5 | VetCompass | Non-neoplastic anal sac disorders in UK dogs: Epidemiology and management aspects of a research-neglected syndrome | 2021 | 10.1002/vetr.203 | 1 | Non-neoplastic anal sac disorders |  |  |  |
| 5 | VetCompass | Keratoconjunctivitis sicca in dogs under primary veterinary care in the UK: an epidemiological study | 2021 | 10.1111/jsap.13382 | 1 | Keratoconjunctivitis sicca |  |  |  |
| 5 | VetCompass | Incidence and risk factors for feline lymphoma in UK primary-care practice | 2021 | 10.1111/jsap.13266 | 1 | feline lymphoma |  |  |  |
| 5 | VetCompass | Development and internal validation of a prediction tool to aid the diagnosis of Cushing's syndrome in dogs attending primary-care practice | 2020 | 10.1111/jvim.15851 | 1 | Cushing's syndrome |  |  |  |
| 5 | VetCompass | Vestibular disease in dogs under UK primary veterinary care: Epidemiology and clinical management | 2020 | 10.1111/jvim.15869 | 1 | Vestibular disease |  |  |  |
| 5 | VetCompass | Clinical management of lipomas in dogs under primary care in the UK | 2020 | 10.1136/vr.105804 | 1 | lipoma |  |  |  |
| 5 | VetCompass | Side Effects to Systemic Glucocorticoid Therapy in Dogs Under Primary Veterinary Care in the UK | 2020 | 10.3389/fvets.2020.00515 | 1 | Side Effects to Systemic Glucocorticoid Therapy in dogs |  |  |  |
| 6 | BristolCats | Owner-reported pica in domestic cats enrolled onto a birth cohort study | 2021 | 10.3390/ani11041101 | 1 | Owner-Reported Pica in Domestic Cats |  |  |  |
| 6 | BristolCats | Influence of living in a multicat household on health and behaviour in a cohort of cats from the United Kingdom | 2020 | 10.1136/vr.104801 | 1 | Influence of living in a multicat household |  |  |  |
| 6 | BristolCats | Owner-reported flea treatment measures and skin disease in cats | 2019 | 10.1177/1098612X18773911 | 2 | Owner-reported flea treatment | skin disease in cats |  |  |
| 6 | BristolCats | Owner-reported lower urinary tract signs in a cohort of young cats | 2017 | 10.1177/1098612X16643123 | 1 | Owner-reported lower urinary tract signs |  |  |  |
| 6 | BristolCats | Risk factors for road traffic accidents in cats up to age 12â€…months that were registered between 2010 and 2013 with the UK pet cat cohort ('Bristol Cats') | 2017 | 10.1136/vr.103859 | 1 | Risk factors for road traffic accidents |  |  |  |
| 6 | BristolCats | Cohort Profile: The 'Bristol Cats Study' (BCS)-A birth cohort of kittens owned by UK households | 2017 | 10.1093/ije/dyx066 | 0 | methodological paper |  |  |  |
| 6 | BristolCats | Risk factors identified for owner-reported feline obesity at around one year of age: Dry diet and indoor lifestyle | 2015 | 10.1016/j.prevetmed.2015.07.011 | 1 | owner-reported feline obesity at around one year of age |  |  |  |
| 7 | Dogslife | The impact of the COVID-19 pandemic on a cohort of Labrador retrievers in England | 2022 | 10.1186/s12917-022-03319-z | 1 | Effects of COVID-19 Pandemic on Puppies |  |  |  |
| 7 | Dogslife | Validity of Internet-based longitudinal study data: the elephant in the virtual room | 2015 | 10.2196/jmir.3530 | 1 | Validity of Internet-based longitudinal study data |  |  |  |
| 7 | Dogslife | Dogslife: A cohort study of Labrador Retrievers in the UK | 2015 | 10.1016/j.prevetmed.2015.06.020 | 3 | height | weight | lifestyle |  |
| 7 | Dogslife | Dogslife: a web-based longitudinal study of Labrador Retriever health in the UK | 2013 | 10.1186/1746-6148-9-13 | 3 | Clinical signs of illnesses | owners actively involved | veterinary presentation |  |
| 8 | Mars Petcare | The MARS PETCARE BIOBANK protocol: establishing a longitudinal study of health and disease in dogs and cats | 2023 | 10.1186/s12917-023-03691-4 | 0 | methodological paper |  |  |  |
| 9 | CaniAge | INTRINSIC CAPACITY ASSESSMENT IN THE CANIAGE PILOT COHORT | 2022 | 10.1007/s12603-022-1772-1 | 4 | Cognitive Dysfunction Syndrome Evaluation tool | memory test | locomotion | vitality |
| 10 | TeamMate | Teammate: A longitudinal study of new zealand working farm dogs. iii. factors affecting the risk of dogs being lost from the workforce | 2021 | 10.3390/ani11061602 | 4 | vaccination status | deworming regime | musculosckeletal abnormalities | bedding/kennel conditions |
| 10 | TeamMate | TeamMate: A Longitudinal Study of New Zealand Working Farm Dogs. II. Occurrence of Musculoskeletal Abnormalities | 2020 | 10.3389/fvets.2020.00624 | 1 | Occurrence of Musculoskeletal Abnormalities |  |  |  |
| 10 | TeamMate | TeamMate: a longitudinal study of New Zealand working farm dogs. I. Methods, population characteristics and health on enrolment | 2020 | 10.1186/s12917-020-2273-2 | 1 | Risk of Dogs Being Lost from the Workforce |  |  |  |
| 11 | WestHighlandWhiteTerrier Cohort | Early-life risk factors and heritability of canine atopic dermatitis: A birth cohort study from West Highland white terriers | 2019 | 10.1111/vde.12812 | 1 | assess early-life risk factors for canine atopic dermatitis |  |  |  |
| 11 | WestHighlandWhiteTerrier Cohort | Natural history of atopic dermatitis in a cohort of West Highland white terriers | 2019 | 10.1111/vde.12812 | 1 | onset of clinical signs of Canine atopic dermatitis |  |  |  |
| 12 | CatPAWS | Dental health in mature cats | 2018 | 10.1177/1098612X18791875 | 1 | dental health in cats |  |  |  |
| 13 | C.L.A.W.S. | Early-life risk factors identified for owner-reported feline overweight and obesity at around two years of age | 2017 | 10.1016/j.prevetmed.2017.05.010 | 2 | feeding practices | feline obesity |  |  |
| 14 | Norwegian large dog | A longitudinal study on the occurrence of <i>Cryptosporidium</i> and <i>Giardia</i> in dogs during their first year of life | 2007 | 10.1186/1751-0147-49-22 | 1 | parasitic infections |  |  |  |
| 14 | Norwegian large dog | A longitudinal study on diarrhoea and vomiting in young dogs of four large breeds | 2012 | 10.1186/1751-0147-54-8 | 2 | vomiting | diarrhoea |  |  |
| 14 | Norwegian large dog | Risk factors for hip-related clinical signs in a prospective cohort study of four large dog breeds in norway | 2012 | 10.1016/j.prevetmed.2011.09.018 | 1 | hip-related clinical signs |  |  |  |
| 14 | Norwegian large dog | The effect of radiological hip dysplasia and breed on survival in a prospective cohort study of four large dog breeds followed over a 10year period | 2012 | 10.1016/j.tvjl.2011.10.015 | 1 | survival |  |  |  |
| 14 | Norwegian large dog | Housing- and exercise-related risk factors associated with the development of hip dysplasia as determined by radiographic evaluation in a prospective cohort of Newfoundlands, Labrador Retrievers, Leonbergers, and Irish Wolfhounds in Norway | 2012 | <https://dx.doi.org/10.2460/ajvr.73.6.838> | 1 | development of hip dysplasia as determined by radiographic evaluation |  |  |  |
| 15 | French Frailty | Canine Model of Human Frailty: Adaptation of a Frailty Phenotype in Older Dogs | 2023 | 10.1093/gerona/glad006 | 2 | time to all-cause death | all-cause death |  |  |
| 15 | French Frailty | Establishing a Frailty Phenotype for Aging Dogs Katharine Russell | 2022 | 10.1111/jvim.16541 | 1 | 6-month mortality |  |  |  |
| 16 | Canadian K9 lifetime (LYME) study | Canadian K9 lifetime (LYME) study: Descriptive analyses of year 1 data | 2018 | 10.1002/jvim.15319 | 7 | seroconversion rate of dogs to B. burgdorferi, Ehrlichia and Anaplasma | incidence of clinical signs of Lyme disease | client perceptions, awareness and education on vector-borne disease |  |
| 17 | Mid Missouri dogs | Three-year longitudinal seroprevalence of vector-borne pathogens in mid-missouri dogs | 2018 | 10.1002/jvim.15319 | 11 | Antibodies (3 pathogens) | seropositive status (7pathogens) | mortality cause |  |
| 18 | Cohort study western Massachusetts | Establishment of a prospective cohort study of domestic dogs in western Massachusetts | 2005 |  | na | Excluded from outcome analysis: only abstract available. | | | |
| 19 | het Boxerproject | A long-term follow up study of a birth cohort of boxer dogs in The Netherlands : genetic and environmental risk factors for diseases |  |  | na | Dissertation |  |  |  |
| 19 | het Boxerproject | Life expectancy in a birth cohort of Boxers followed up from weaning to 10 years of age | 2005 |  | 1 | Life expectancy |  |  |  |
| 19 | het Boxerproject | Genetic and epidemiological investigation of a birth cohort of boxers | 2003 |  | 2 | study mortality | disease incidence |  |  |
| 20 | Agria Pet Insurance | The epidemiology of stifle joint disease in an insured Swedish dog population | 2021 |  | 1 | stifle joint disease |  |  |  |
| 20 | Agria Pet Insurance | The epidemiology of cruciate ligament rupture in an insured Swedish dog population | 2021 |  | 1 | cruciate ligament rupture |  |  |  |
| 20 | Agria Pet Insurance | The epidemiology of upper respiratory tract disorders in a population of insured Swedish dogs (2011-2014) and its association to brachycephaly | 2023 |  | 1 | upper respiratory tract disorders |  |  |  |
| 21 | Pandemic Puppies | Pandemic Puppies: Demographic Characteristics, Health and Early Life Experiences of Puppies Acquired during the 2020 Phase of the COVID-19 Pandemic in the UK | 2022 |  | 3 | puppy early-life behaviour, | socialisation/habituation | health |  |
| 21 | Pandemic Puppies | Is UK Puppy Purchasing Suffering a Long COVID Effect? Ongoing Negative Impacts of the COVID-19 Pandemic upon Puppy Purchase Motivations and Behaviours in 2021 | 2023 |  | 2 | Demographic Characteristics, Health and Early Life Experiences of Puppies |  |  |  |
| 22 | SAVSNET |  |  |  |  | Excluded from outcome analysis: no paper identified in the literature search | | | |
