# Supplementary material for: A mapping review of worldwide current and previous cohort research programmes in cats and dogs
Source: PLoS One. 2025 Jun 2;20(6):e0321007. doi: 10.1371/journal.pone.0321007 (PMC12129338; doi:10.1371/journal.pone.0321007)
Supplement: Attachment — Submitted filename: Response to Reviewers.docx [file pone.0321007.s009.docx]

**Dear Reviewer’s of PlosOne**

We sincerely thank you for your thorough review of our manuscript titled “A Mapping review of worldwide current and previous cohort research programmes in cats and dogs.” We greatly appreciate the time and effort you have invested in providing valuable feedback.

We have carefully addressed all the points raised and have revised the manuscript accordingly. Below, we provide detailed responses to each of your comments, highlighting the specific changes made.

Thank you once again for your constructive input and for helping us enhance the manuscript.

**Sincerely,**
Dr. Malwina Kowalska
On behalf of all authors

Journal Requirements:

| ***Comment*** | ***Response*** | ***Page/Line*** |
| --- | --- | --- |
| 1) Ensure that your manuscript meets PLOS ONE's style requirements | Thank you. Manuscript was adjusted to the PLOS One Style. |  |
| 2) Please note that, though access restrictions are acceptable now, your entire data will need to be made freely accessible if your manuscript is accepted for publication. This policy applies to all data except where public deposition would breach compliance with the protocol approved by your research ethics board. If you are unable to adhere to our open data policy, please kindly revise your statement to explain your reasoning and we will seek the editor's input on an exemption. Please be assured that, once you have provided your new statement, the assessment of your exemption will not hold up the peer review process. | Thank you for your comment. That was a mistake on my side. Data was and is free available at the OSF, which is indicated in the Acknowledgments section: “The data collection template is available in S2 File and at the OSF; data used for all analyses, analytic R code, and screener instructions are available at the OSF (<https://osf.io/wkg53/>).” |  |
| 3) Please include captions for your Supporting Information files at the end of your manuscript, and update any in-text citations to match accordingly. Please see our Supporting Information guidelines for more information: http://journals.plos.org/plosone/s/supporting-information. | Thank you. A supporting information section was included. Cytation and naming were done according to the PLOS One guidelines. |  |
| 4) We note that there is identifying data in the Supporting Information file Supplementary File 2.xlsx. Due to the inclusion of these potentially identifying data, we have removed this file from your file inventory. Prior to sharing human research participant data, authors should consult with an ethics committee to ensure data are shared in accordance with participant consent and all applicable local laws | Thank you very much for a detailed information. Authors names and Institution location was removed from a list of records that entered stage II of the selection in the mapping review. |  |
| 5) Please review your reference list to ensure that it is complete and correct | The list was reviewed and formatted. No cited papers have been retreated or added to the list. |  |

## Reviewer 1

| ***Comment*** | ***Response*** | ***Page/Line*** |
| --- | --- | --- |
| The article provides a systematic overview of cohort research programs involving cats and dogs. Using a mapping review methodology, the study identifies 22 programmes, primarily in the UK and US, focusing on topics like preventive medicine, geriatrics, and animal behavior. Key findings highlight gaps in study design reporting, funding transparency, and data management practices. The review emphasizes the need for better reporting standards, pre-registration practices, and increased collaboration to improve the quality and global reach of veterinary cohort research. The article also reports that the majority of cohort research programs (approximately 50%) are devoted to preventive medicine, although this detail is not explicitly mentioned in the article’s summary. Including this percentage, would enhance the clarity of findings. For instance, reporting that 11 out of 22 cohort programs focused on preventive medicine highlights this key insight more transparently. | Thank you for the comment. We expanded the abstract text to highlight and include additional information about the focus of identified cohort programmes. | Line 42-43 |
| Additionally, the main conclusions of these cohort research programs could be summarized to reflect common goals such as disease prevention, monitoring of health outcomes, and understanding environmental and genetic factors influencing companion animal health. Including such conclusions in the summary would provide a more comprehensive overview of the research's contributions to veterinary medicine | Thank you for this valuable suggestion. We have adjusted the conclusions section to summarize the common goals of the cohort research programmes, including understanding environmental and genetic factors influencing companion animal health. | 724-725 |
| L123-L125: Could you please improve the syntax of these lines? | Thank you for your suggestion to improve the syntax. We have revised the sentence for clarity as follows:  'In this mapping review, we specifically focused on cohort research programs involving only cats or dogs, which we will refer to as "programmes." | 132-134 |
| L127. In the paper context what does it mean demographics? | Thank you for your comment. To make it more cleare we add more examples: “In the eligible programmes, animals are enrolled primarily based on demographics (e.g., cat or dog species, age, sex, breed) rather than health status (e.g., cancer registries)” | 137 |
| L209. Term "guardian of pets" is increasingly used instead of "owner" as it emphasizes responsibility, care, and protection. It aligns with a more compassionate and ethical approach to human-animal relationships. | Thank you for the valuable comment. We left “owner” in the phrase “owner consent forms” as we believed this is still a commonly used term and functioning as a whole phrase. In any other places, text was adjusted according to your suggestion. | 189, 226, 237, 426  440, 482, 486, 488  515, 526, 528 |
